# MECHANISMS IN ENDOCRINOLOGYEndocrine and immunological aspects of burnout: a narrative review

**DOI:** 10.1530/EJE-18-0741

**Published:** 2018-12-20

**Authors:** Ingibjörg H Jonsdottir, Anna Sjörs Dahlman

**Affiliations:** 1 Institute of Stress Medicine, Region Västra Götaland; 2 Department of Food and Nutrition, and Sport Science, University of Gothenburg, Gothenburg, Sweden

## Abstract

Burnout has several different definitions, and attempts have been made to discriminate between burnout as a psychological construct and burnout as a clinical entity. A large body of research has focused on elucidating the biological link between stress exposure and burnout and/or finding a clinically usable biomarker for burnout. The objective of this narrative review is to summarize the main endocrine and immune findings in relation to burnout. The literature has primarily focused on dysregulation of the hypothalamus-pituitary-adrenal (HPA) axis. However, albeit the large body of studies, it cannot be concluded that clear effects are seen on HPA axis function in people with burnout. The HPA axis and anabolic acute reactivity to stress might be affected in clinical burnout. Plausible, effects of chronic stress might rather be seen when measuring responses to acute stress rather than resting state hormonal levels. Studies on other hormones, including thyroid hormones, prolactin and growth hormone in burnout subjects are inconclusive. It is important to note that this field is faced with many methodological challenges, one being the diurnal and pulsatile nature of many of the hormones of interest, including cortisol, which is not always considered. Another challenge is the heterogeneity regarding definitions and measurements of stress and burnout. Existing studies on burnout and immune function are heterogeneous regarding the results and no firm conclusion can be made if clinically relevant immune changes are present in burnout subjects. An overall conclusion is that existing research cannot confirm any homogenous reliable endocrinological or immunological changes related to burnout.

## Invited Author’s profile

**Prof. Ingibjörg Jonsdottir** is a director for the Institute of Stress Medicine (ISM), a practitioner-oriented research institute in Sweden working with all aspects of stress-related mental health including both healthy workplaces, particularly the organizational perspective but also clinical research on patients with exhaustion/burnout. She has a PhD in physiology and is affiliated as a professor at Gothenburg University, Gothenburg, Sweden.


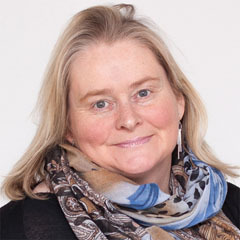


## Introduction

The consequence of chronic sress is becoming a major research topic combining various research disciplines including psychology, sociology and physiology. Ever since Hans Selye wrote the important work about a stress condition known as ‘general adaptation syndrome’, the research on environmental stress and stress responses has developed tremendously. His early assumption, however, that the physiological stress response is a general and non-specific reaction to any environmental stressor has now been further developed (1). Today, we know that the stress reaction in humans is far more complicated than originally described in animal research. Stressful situations are multifaceted involving complicated cognitive processes resulting in different appraisal and coping by each individual and situation (2, 3). In fact, the stress response involves several systems, the physiological reaction being one of them. Cognitive, emotional and behavioral responses are also important for the outcome of the overall stress response. Furthermore, we also know that the physiological stress reaction involves several endocrine systems that are closely linked to each other, including the hypothalamic–pituitary–adrenal (HPA) axis and the hypothalamic–pituitary–thyroid (HPT) axis. The physiological stress response also triggers complex immunological responses, as well as the release of both catabolic and anabolic hormones (4, 5).

Thus, expecting that all individuals will show the same or similar outcome during acute stress and that pure relation is seen between the magnitude of appraisal and the physiological response is too simplistic. For instance, significant correlations between cortisol responses and perceived emotional stress variables were found in merely 25% of studies on acute stress and cortisol reaction (6). Various elements that potentially could contribute to this apparent dissociation include both methodological features of the stress protocols and interindividual differences regarding the psychological and emotional appraisal. Kudielka *et al*. (7) also elaborated on factors explaining why we respond differently to stress. These include early life experiences, social factors, psychological interventions, personality as well as acute subjective-psychological stress responses and the states of chronic stress and psychopathology. Furthermore, the endocrine systems most often studied in stress research show diurnal variations and pulsatile release of hormones, which introduces additional challenges regarding interpretation of the measurements. Considering that the acute stress reaction differs substantially between individuals and that the stress response cannot be considered universal, it becomes even more complicated to study objective biological findings related to chronic psychosocial stress. It has clearly been shown that chronic perceived psychosocial exposure is an important contributor to several health impairments including cardiovascular disease and depression (8, 9). It is therefore understandable that many researchers seek a physiological pathway that plausibly could explain the link between stress and disease. However, it is also clear the all individuals do not develop health problems as a consequence of high levels of perceived stress (10). Thus, the characteristics of the stress exposure and various resilience and vulnerability factors could potentially influence the health outcome, and plausible physiological correlates could vary substantially between different individuals.

### Burnout: many different definitions and faces

The initial papers on burnout were published during the mid-1970s by Freudenberger followed by Maslach and coworkers (11, 12). The term originates from the social and work psychology describing symptoms of emotional depletion and loss of commitment and motivation in people working with patients or clients. Originally, burnout research dealt with interpersonal relationships between the worker and the patient/client and not so much about the individual stress response or plausible health consequence (13). During the 1980s, other researchers further elaborated on the theoretical basis of burnout and different definitions and constructs arose (14, 15). In the most widely used burnout construct originally defined by Maslach (13), burnout is described as having three key dimensions – an overwhelming emotional exhaustion (EE), depersonalization (DP) and a sense of ineffectiveness and lack of personal accomplishment (PA). The burnout construct defined by Shirom and Melamed described the term as a chronic depletion of an individual’s energetic resources due to chronic stress. In their conceptualization, burnout consists of the three dimensions: physical fatigue, EE and cognitive weariness, which differs considerably from Maslach’s definition (13, 16). Thus, Shirom and Melamed argued that depersonalization and diminished personal effectiveness may not necessarily be associated with the individual’s feelings of being emotionally exhausted, physically fatigued and cognitively worn-out. However, all constructs of burnout, despite several dissimilarities, emphasize exhaustion as the main component of the burnout syndrome (17).

Initially, burnout was referred almost exclusively to people-oriented professionals (e.g. teachers, nurses, doctors, social workers and police enforcements) (13). We now know that other working populations as well as elite athletes and parents of chronically ill children can suffer from burnout (18, 19). Thus, burnout defined as symptoms of exhaustion due to long-term exposure to any situation that is emotionally demanding cannot be defined as solely work related (18, 19, 20, 21).

A development toward medical research disciplines studying the somatic health consequences was seen during the 1990s initiated by Melamed and coworkers (22, 23). Increased risk of cardiovascular disease (CVD) has been clearly linked to both chronic work-related stress (24) as well as vital exhaustion (25), which is closely related to burnout. Only two prospective studies have been identified relating CVD to burnout and only one of them used a validated burnout scale showing that burnout is a significant risk factor for development of CVD (26, 27). Burnout has also been linked to other health consequences including musculoskeletal pain and metabolic disturbances (28).

### Burnout as a clinical diagnosis

Already during the mid-1980s, it was suggested that a distinction should been made between burnout as a work-related stress syndrome and burnout as a clinical mental problem (29, 30). Typically, patients seeking health care for exhaustion due to long-term stress exposure reported longstanding fatigue, sleep impairments and problems with memory and concentration as their chief complaints, and these characteristics are different from the initial definition of burnout (31).

Attempts have been made to adapt the burnout concept to be more usable in clinical practice. The initial theoretical definition of the burnout construct might not be suitable for clinical practice and indeed it has been shown that the most utilized burnout tool, the Maslach burnout inventory (MBI), was not suitable to be used as a diagnostic tool for patients (32). In the Netherlands, clinical burnout as a clinical diagnosis has been suggested, using the diagnostic criteria of neurasthenia, adding the component that the problem should be work related (17). The clinical diagnosis ‘exhaustion disorder’ (ED) has been proposed by the National Board of Health and Welfare in Sweden to be used in clinical practice. ED defines patients with exhaustion that has developed because of identifiable stressor(s) that have been present for at least 6 months (31). The symptoms of ED and burnout are closely related, and most patients fulfilling the diagnostic criteria for ED can also be described as burned out (33, 34).

### The pathophysiology of burnout

A large body of research has focused on elucidating the pathophysiology of burnout with the goal of finding diagnostic biomarkers for clinical burnout or to search for a biological link between environmental stress exposure and the development of burnout. Research studying the plausible pathophysiological mechanisms has primarily focused on dysregulation of the HPA axis and immune functions (35). Below, we summarize the main endocrine and immune findings in clinical and non-clinical burnout populations.

## Methods

We searched MEDLINE, Scopus and Web of Science for English-language articles containing the keyword *burnout* together with each of the following keywords: *hormone*, *biomarker*, *endocrine*, *endocrine system*, *HPA axis*, *thyroid*, *immune*, *immune system*, *immunological*, *inflammation*, *inflammatory*. We included articles that described the study population in terms of burnout (clinical or non-clinical) and reported measurements of one or more biological analyte that could be categorized as either an endocrine or an immune marker. In a second round of search, the keyword burnout was entered together with the names of endocrine and immune markers identified in the first round of search (i.e. cortisol, DHEA, ACTH, adrenaline/epinephrine, prolactin, testosterone, progesterone, estradiol, TSH, thyroxin, triiodothyronine, growth hormone, oxytocin, cytokine, CRP, leukocyte). From the articles retrieved, additional references were identified by a manual search among the cited references. Articles published until August 2018 were included in this review.

## Endocrine function and burnout

### HPA axis and burnout

Historically, the HPA axis has been the most common endocrine focus in burnout research. This is logical since the HPA axis together with autonomic nervous system are the two key components of the acute stress reaction responsible for mobilizing a successful adaptive response to different stressors. However, the HPA axis function in burnout has mainly been evaluated in terms of cortisol awakening response (CAR) and diurnal cortisol. Heterogeneity in the literature linking burnout to the CAR is the rule rather than the exception. Most studies have not been able to show a significant difference in CAR between clinical populations of burned-out subjects and controls (36, 37, 38, 39). One study found elevated morning salivary cortisol levels among female burnout patients (40), whereas another study found indications of a smaller CAR in male burnout patients, but the difference appeared to be mainly related to the antidepressant use (41). De Vente *et al*. (42) found no difference in the response after awakening, but burnout patients showed elevated cortisol levels during the first hour after awakening in comparison to healthy controls. In a study by Mommersteeg *et al*. (43) burnout cases had significantly lower morning cortisol levels, but the rise after awakening was similar in the burnout and control group. This heterogeneity may result from inconsistency in how CAR is measured (44) and from failure to control for confounding factors. Sleep problems are common in burnout but very few studies have assessed sleep quality and quantity the night before cortisol sampling. Disturbed sleep and multiple awakenings could influence the CAR (45).

Previous studies measuring diurnal salivary cortisol levels have reported no difference in diurnal cortisol between burnout cases and healthy controls (37, 41, 46, 47) or decreased daytime and/or evening cortisol (38, 48), whereas elevated daytime cortisol levels has been related to burnout in other studies (49, 50, 51). The time point of measurements does matter as a couple of studies have found relationships between burnout and awakening cortisol (albeit in opposite directions) but found no relation to burnout when cortisol was measured later in the day (52, 53). This raises a major methodological issue related to this field of research, namely the importance of handling the circadian variations in the study design. There are also naturally occurring ultradian variations as well as superimposed secretion peaks due to physical or psychological stress factors that should be taken into consideration.

Measuring HPA axis function when the stress system is challenged might be a more sensitive method to reveal chronic stress-related alterations. This has been studied by several research groups, but heterogeneous results are reported here as well. Several authors have reported that dexamethasone suppression tests do not differ between burnout patients and controls, indicating sustained negative feedback sensitivity in burnout patients (36, 37, 54). Attenuated HPA axis responses to the combined dexamethasone and corticotropin-releasing hormone (DEX-CRH) challenge has been reported in women on long-term sick leave with job stress-induced depression, a patient group very similar to clinical burnout patients (55, 56). Similarly, some studies of negative feedback in healthy workers have shown an association between high burnout scores and stronger suppression (46, 57). In healthy school teachers, plasma cortisol responses to synthetic ACTH (Synacthen) stimulation were related to EE but not to overall burnout score or the other two subscales of the MBI (58).

The ability to mobilize adequate physiological responses to acute stressors has also been studied in patients with stress-related conditions. A few studies have investigated HPA axis responses to the Trier Social Stress Test (TSST) and modified TSST. De Vente *et al*. found no deviations in HPA axis reactivity and recovery during and after acute stress in one study (42) and lower cortisol reactivity in males in another study (59). In the study by Lennartsson *et al*. (60), there were no overall differences in responses of ACTH, serum cortisol or salivary cortisol between patients and controls. However, patients reporting higher burnout scores had lower salivary cortisol responses than controls, indicating that patients with more severe burnout symptoms may be hypocortisolemic in their response to acute stress. A trend to lower ACTH responses was also seen. Similar results were reported by Jönsson *et al*. (61); former patients still scoring high burnout had a blunted HPA axis response to a virtual TSST compared to healthy controls and recovered former patients.

This research area has been dominated by the assumption that a HPA dysregulation will be seen in chronic stress conditions, including burnout and exhaustion. This does, however, not really seem to be the case, since the results show heterogeneous results, and in fact, most studies do not confirm HPA axis dysregulation in burnout patients. Cadegiani and Kater (62) recently performed a systematic review of adrenal function, including HPA axis function, in burnout and found an almost systematic finding of conflicting results. If measured correctly, cortisol is an excellent indicator of acute stress reactions, whereas its applicability as a marker of chronic stress is highly questionable. Basal cortisol measures such as CAR or diurnal cortisol cannot be concluded to be generally affected in burnout patients, and these measures are questionable given the large circadian and ultradian variations. Measurement of HPA axis function when the stress system is challenged, either pharmacologically or during psychosocial stress, also show heterogeneous result. However, Cadegiani and Kater (62) argue that the most appropriate methods to assess the HPA axis have not been used in burnout studies and functional tests, such as the insulin tolerance test, should be employed. Table 1 summarize the HPA findings in clinical and non-clinical burnout showing that it cannot be concluded that clear effects are seen on HPA axis function in people with burnout. The HPA axis reactivity might be affected in patients with clinical burnout/exhaustion, but more studies are needed to confirm this.

**Table 1:** Summary of HPA axis findings in clinical and non-clinical burnout.

HPA axis measure	Higher than controls or positive relationship with burnout symptoms	Lower than controls or negative relationship with burnout symptoms	No difference between groups or no relationship with burnout symptoms
CAR		(52)	(36, 37, 38, 39, 41, 42, 43, 48, 49, 63)
Morning cortisol level	(40, 42, 49, 53)(47) on workdays	(43, 64, 65)	(38, 64, 66)
Diurnal cortisol variation		(65)	(37, 41, 46, 52)
Daytime/evening cortisol level	(49, 50, 51, 65)	(38, 48)	(47, 53, 67)
Urine free cortisol, 24 h		(64)	
Cortisol after DEX (or DEX/CRH)		(46, 55, 56, 57)	(36, 37, 54, 63)
Response to acute stress		(59) Males(60, 61) more severe cases	(42, 60)

### Anabolic hormones and burnout

The physiological stress reaction also includes the release of anabolic hormones with protective and regenerative roles (68). Similar to the HPA axis, the theoretical background for studying anabolic hormones in burnout is the search of a biological link between stress and disease (69). Few studies, however, have focused on the anabolic part of the endocrine system in people with burnout.

**Figure 1:**
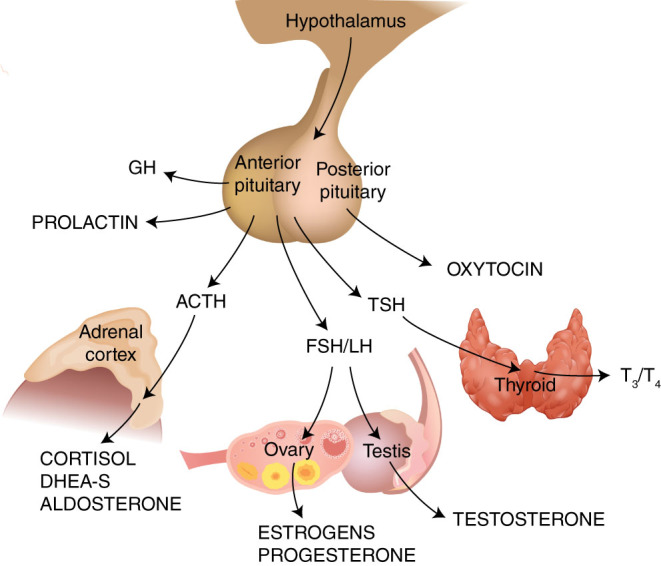
Major endocrine systems studied in relation to burnout.

The most frequently studied anabolic hormone is dehydroepiandrosterone (DHEA). Both DHEA and its sulfated metabolite (DHEA-S) are androgen precursors that, as cortisol, are secreted by the adrenal cortex in response to ACTH (Fig. 1). DHEA and DHEA-S have been shown to have neuroprotective, antioxidative, anti-inflammatory and antiglucocorticoid effects (70, 71). Levels of DHEA-s are known to temporarily increase during acute psychosocial stress (72, 73) and the acute stress-induced DHEA-s release has been suggested to play a protective role, as an antagonist to the consequences of cortisol (72). One study comparing baseline DHEA-s between burnout subjects and healthy controls reported higher DHEA-s levels in the burnout subjects (36), whereas three other studies reported no differences in baseline DHEA-s levels between burnout subjects and controls (63, 64, 66). In a study on baseline DHEA-s levels in patients with burnout in different age groups, it was found that low DHEA-s levels were present only in the younger patients (25–34 years) (74). Moreover, DHEA-s production capacity during acute stress was shown to be attenuated in patients with clinical burnout (75) and increased DHEA-s levels during the first year of treatment has been linked to better health development in burnout patients (76). Thus, DHEA-s response to challenge in burnout patients and the relation to health development and recovery deserves further investigation.

The HPA axis and the hypothalamic–pituitary–gonadal (HPG) axis are competitive systems and during prolonged periods of stress, the HPG axis and the production of sex steroids could be inhibited (68). Testosterone, progesterone and estradiol levels have also been investigated in relation to burnout. Åsberg *et al*. (77) found some indications of increased testosterone levels in women on long-term sick leave for a stress-related affective disorder. Hitherto, no significant alterations in progesterone or estradiol levels have been reported (66, 78).

### HPT axis and burnout

The core dimension of burnout is exhaustion and thus all hormonal system involved in mobilizing energy are of interested to study in subjects with burnout. Compared to the focus on the HPA axis, the HPT axis has received much less attention in the burnout literature. The HPT axis is a central regulatory system that controls the production of thyroid hormones. Hypothalamic thyrotropin-releasing hormone stimulates the synthesis and secretion of pituitary thyroid-stimulating hormone (TSH), which in turn acts at the thyroid to stimulate thyroxin (T4) and triiodothyronine (T3) biosynthesis and secretion. Acute stress has been shown to causes transient activation of the HPT axis, whereas prolonged stress is associated with decreased activity (79, 80). 

Two studies have reported normal levels of T4, T3 and/or TSH in burnout cases compared to non-burnout groups (81, 82). Significantly lower TSH and T3 levels have been found in female populations reporting stress-related mental health problems (77).

Investigations of possible HPT axis alterations in burnout are problematic, partly because of the overlapping symptoms between thyroid diseases, especially hypothyroidism and burnout syndrome and partly because of patients included in burnout studies. Deviating thyroid hormone levels is often an exclusion criterion in clinical burnout studies. Therefore, the true prevalence of HPT axis deficiencies in clinical burnout and the causal relationship between plausible HPT alterations and burnout is difficult to determine.

### Other endocrine systems and burnout

Several other hormones and growth factors have been studied in various burnout populations with the goal of elucidating the pathophysiology of burnout and to find diagnostic biomarkers for clinical burnout.

One anterior pituitary hormone that has been shown to increase in response to different types of psychosocial stressors is prolactin. Since burnout is a consequence of long-term psychosocial stress, prolactin levels might also be affected in burnout. Prolactin levels have been studied predominantly in women with burnout. Most of these studies have reported no differences in prolactin levels between women with burnout and control groups (64, 66, 77). Tops *et al*. (67) compared with prolactin levels in nine female burnout subjects with nine female healthy controls. The authors reported that there was an extreme distribution of prolactin levels among the burnouts; some had higher levels than the controls and some had lower levels than the controls. They also reported that female burnout cases with low prolactin had low oxytocin levels. In the study by Åsberg *et al*. (77), significantly lower prolactin levels were found in women on long-term sick leave for a stress-related affective disorder compared to healthy subjects, whereas working women scoring high burnout did not differ from controls. These studies did not adequately control for menstrual cycle phase and use of oral contraceptives or hormonal replacement, which makes data difficult to interpret since prolactin levels are dependent of estradiol levels. One study investigated prolactin levels in both men and women (78). Men who reported burnout had markedly higher prolactin levels than non-burnout controls. Prolactin levels in women in the follicular phase of the menstrual cycle did not differ between burnout and non-burnout groups. The biological significance of prolactin in relation to stress and burnout is not fully known but previous research indicates that prolactin has a protective role against the negative consequences of stress (83, 84).

Moch *et al*. (64) performed a study comparing 16 female burnout patients with 16 female healthy age-matched controls over the course of a 4-month stress management intervention. In addition to measuring cortisol, ACTH, DHEA-s and prolactin already mentioned above, they measured aldosterone, growth hormone and catecholamines (epinephrine and norepinephrine). Growth hormone, aldosterone and epinephrine were not significantly different between patients and controls, whereas norepinephrine was lower in patients compared to controls at baseline. No clear conclusions can be drawn from the mixed findings in this small study. Again, methodological considerations regarding measurements of various hormones are relevant to raise. For instance, the secretion of growth hormone is pulsatile and varies both during the day and in response to different factors such as food intake and to physiological stresses, wherefore single measurements are problematic. The secretion of epinephrine and norepinephrine is highly dependent on various stressors, and the very short half-life of this hormone makes single measurements inappropriate for scientific use in the evaluation of stress. 

## Immune function and burnout

Chronic psychological stress has been suggested to alter immune function and many studies have been undertaken to identify immunological diagnostic or prognostic biomarkers or to explore linking pathways between the neuroendocrine and immune system in chronic stress-related diseases (85). However, there are only a few studies that specifically have evaluated immunological processes in burnout.

### C-reactive protein

C-reactive protein (CRP) is commonly used as a marker of general inflammation, and it has been proposed that chronic low-grade inflammation, as indicated by slightly to moderately increased CRP levels, is associated with chronic stress (86). Studies on CRP levels in burnout cases have shown contradictive results, with some studies showing no differences between burnout and non-burnout subjects (66, 87), whereas others reported increased CRP levels related to burnout (34, 82). Toker *et al*. (88) reported a positive association between CRP levels and higher burnout scores in women, but not in men. To summarize, it remains unclear if burnout syndrome is associated with increased low-grade inflammation as measured by CRP and/or if there are gender differences.

### Leukocyte numbers in blood

A few groups have investigated the association between the absolute numbers of different white blood cell (leukocyte) types and burnout symptoms. Metlaine *et al*. (82) found higher mean leukocyte, neutrophil and monocyte numbers in burnout white-collar workers as compared with healthy controls, whereas other studies did not find any association between different lymphocytes subsets and burnout (36, 89, 90).

To summarize, burnout does not seem to be associated with large changes in number of leukocytes in the blood.

### NK cell numbers or activity

Emotional stress has been reported to be associated with decreased NK cell activity in animals and in humans (85, 91). NK cell numbers and activity have been the focus of a few studies in burnout. Nakamura *et al*. (92) reported an association between high burnout depersonalization subscale score and decreased NK cell activity in male office workers, whereas no correlation between NK cell activity and EE or PA scores was found.

No relation between absolute NK cell numbers and burnout was found in the studies by Bargellini *et al*. and Mommersteeg *et al*. respectively (36, 89).

Further studies are needed to elucidate if burnout has any effects on the number and/or function of different blood leukocyte subsets, including T, B and NK cells. In addition, it remains unknown if a change, for example, reduction, of cellular immunity has any clinical relevance such as increased risk of infections or malignancies.

### Cytokines

The association between different cytokines, as measured in plasma from blood samples or after *in vitro* stimulation of isolated leukocytes, and burnout has been evaluated in a handful of studies. Both levels of individual cytokines as well as ratios between pro-and anti-inflammatory cytokines have been studied in relation to burnout.

High burnout scores were associated with higher TNFα levels in plasma, whereas no relation between burnout score and TGFβ was seen in the study by Grossi *et al*. (66).

Gajewski *et al*. (90) measured 17 different cytokines, including TNFα, in blood and found no relation between any of the cytokines and EE or depression when analyzing their entire study population. However, the plasma concentrations of the pro-inflammatory cytokines IL-6 and IL-12 were positively correlated with the extent of EE in men but not in women.

No significant differences were seen for any of the cytokines IL-1β, TNFα, IL-2, IL-6, IL-8, MCP-1, EGF and VEGF between women with exhaustion due to prolonged psychosocial stress and healthy controls (34), whereas Åsberg *et al*. found increased levels of MCP-1, EGF and VEGF, but no changes in cytokines levels in women exposed to prolonged psychosocial stress (77). Plausible reasons for the discrepant findings may be the large interindividual variation of cytokine levels, intra-individual (e.g. circadian) variation, the different assays and selected cut-offs used.

Higher total burnout scores predicted higher plasma TNFα levels, lower IL-4 levels and a higher TNFα/IL-4 ratio in a study in working school teachers (93), with a reservation of interpolated values for IL-4 due to problems with detection limits. No association was found between IL-10 levels or TNFα/IL-10 ratios and burnout scores.

One study assessing the *in vitro* cytokine production after stimulation of whole blood found that the IL-10, but not TNFα, release in response to LPS stimulation (mainly stimulating monocytes) was higher in burnout than in healthy controls but no difference in IL-10 or IFNγ were seen after PHA (T cell stimulator) were seen (36). The *in vitro* suppressive effect of dexamethasone on PHA stimulated IFN or IL-10 production or LPS-stimulated TNF or IL-10 production was similar in burnout and healthy controls.

Summarizing the research on burnout and cytokines, no conclusion can be made regarding plausible relationship between burnout and cytokines levels, partly because few studies have been conducted but mainly due to discrepancy among existing studies.

## Discussion

The burnout construct is a complex phenomenon that over time has been differently defined (94). Burnout research includes both working populations reporting high levels of fatigue/exhaustion as well as clinical populations with clear symptoms of exhaustion due to various long-term stress exposure and insufficient recovery. Furthermore, the theoretical background and consequently the methods used to measure burnout differ considerably between studies. Exploring endocrinological and immunological functions in relation to burnout is thus associated with certain challenges. Indeed, these challenges can be extrapolated to the whole field of stress research, since definitions and methods used to measure psychosocial stress differs enormously between studies. It is understandable that the largest research focus has been on the HPA axis, this being one of the key components of the acute stress reaction responsible for mobilizing a successful adaptive response to various stressors. Plausibly, the utmost reason for the large focus on the HPA axis is the interest in investigating the biological pathways linking stress and disease. Another reason is the urge to find a validated objective manifestation and/or biomarker of stress-related conditions, usable for diagnostic purposes and/or to monitor the course of illness over time. However, this area of research is suffering from a lack of endocrinological knowledge as many studies do not consider the diurnal, pulsatile or the individual variation of the hormonal levels measured.

The hormonal and metabolic changes due to chronic stress seem to be highly individual, contextual and relatively small. Thus, although the results may be statistically different from what is seen in healthy persons, they are most often within normal ranges and thus not clinically relevant. These hormonal measurements cannot be used, neither in the definition nor for diagnosis of burnout. Furthermore, to make firm conclusion of plausible changes related to a disease state, a relatively homogenous patient population is required, including factors such as stress exposure, stress reactions, sleep patterns and symptom characteristics. This is rarely the case for stress-related conditions, including burnout. For instance, the idea that burnout is always preceded by a period of the classical physiological stress arousal might not always be the case since the process of developing burnout can differ considerably. The biological state preceding the development of burnout might be different in people developing burnout as a consequence of caring for a sick relative compared to those developing burnout due to high demands at work (21). Another important aspect when studying the plausible effects on endocrine systems, particularly the HPA axis, is sleep patterns, and this is often difficult to control for (7, 62). We can also speculate that some publications bias might be present, that is, a certain amount of studies showing non-significant results might not have been published. Kakiashvili and coworkers suggested in a review that evaluation of the HPA axis in suspected burnout cases should be brought to the attention of primary care physicians. Although some studies have reported striking statistically significant results, advocating for the use of salivary cortisol measurements in the assessment and biomonitoring of burnout (49), these results are clearly not generalizable when the whole body of research is taken into consideration. Thus, our conclusion is in line the conclusion made by Cadegiani and Kater that it cannot be concluded that adrenal fatigue is present in subjects with burnout and that cortisol should not be used as a marker for burnout syndrome by health practitioners (62). An exception are the few studies available studying HPA axis reactivity in response to challenge, which might be affected in patients with clinical burnout/exhaustion, but more studies are needed to confirm this. This raises an important issue not always considered in stress research; that is, the important discrimination between measuring basal hormonal and/or immunological markers compared to studying biological responses to challenges.

In the search of an explanatory link between stress and disease, the excessive focus on HPA axis studies might have influenced the interest of studying other endocrine systems in relation to burnout. Anabolic hormones are of utmost importance for health and well-being, but few studies have focused on the anabolic processes in people with burnout. Like the HPA axis studies, heterogeneous results are shown when for example DHEA and/or DHEA-s has been measured in relation to burnout. Interestingly, DHEA-s production capacity during acute stress may be attenuated in patients with clinical burnout (75). This, again, raises the question of focusing more on the plausibility that pathophysiological effects of chronic stress could rather be seen when measuring people’s ability to mobilize energy when responding to acute stress, than when measuring hormonal level in a resting situation. The principle of using various challenge tests, such as glucose tolerance tests and exercise ECG, is well known from other medical disciplines. Moreover, studies on other endocrine hormones, including thyroid hormones, prolactin and growth hormone in burnout subjects are inconclusive, mainly due to few studies, mixed findings and small samples.

Similar reasoning as above can be used when discussing immune function. Studying the basal levels of immune cells or cytokines differs from functional tests, and it is unclear if a change, for example, reduction, of cellular immunity has any clinical relevance such as increased risk of infections or malignancies. One of the important characteristics of the immune system is the large redundancy with overlapping functions and effects between immune cells and cytokines. Thus, due to this redundancy, an observed change for one cell type or cytokine may be unlikely to have clinical relevance, that is, increased risk for infections or malignancy. Furthermore, studying many immune parameters simultaneously, for example, both cytokines and cell types can result in mass significance.

It is understandable that immune function has been in focus for many researchers working with stress and burnout. From an evolutionary perspective, immune responses to stressful situations may have been beneficial for survival by increasing the ability to fight off wound infections and promoting wound repair. Indeed, several physiological connections exist between the nervous system/HPA axis and immune organs and cells but generally studies on burnout and immune markers lack theoretical discussion of the relation between the peripheral measured biomarker such as CRP and leukocyte and the mental symptoms of burnout. 

However, summarizing the existing literature, no clear conclusion can be made if immune function is altered in burnout subjects.

Some additional methodological considerations related to this field of research should be mentioned. Many studies in this field have small sample sizes (*N* < 100), which increases the risk of misestimation of both the magnitude and the direction of any true underlying effect (95). The most prominent methodological issue within the field, however, is the heterogeneous definition of the psychological construct burnout that encompasses both working population as well as clinical populations with clear symptoms of exhaustion. Plausible, pathophysiological mechanisms could thus differ considerable depending on the population studied.

## Conclusion

An overall conclusion is that existing research cannot confirm any homogenous reliable endocrinological or immunological changes related to burnout. A consensus definition of burnout is needed to enable the elucidation of the biological link between stress and disease and/or the search of a biological marker for burnout with plausible application in clinical practice. Finding a pathognomonic sign of burnout might not be a realistic goal, but to further investigate the pathophysiology of burnout and to delineate burnout from other mental and stress-related disorders remains an important avenue for future research. Furthermore, the methodology employed to evaluate the proposed correlation between burnout and endocrine and immune function should be standardized. An important concluding remark is that HPA axis hormone measurements are not suitable to use for diagnostic purposes in routine clinical practice, i.e., primary care or occupational health services for patients with stress-related conditions such as burnout.

## Declaration of interest

The authors declare that there is no conflict of interest that could be perceived as prejudicing the impartiality of this review.

## Funding

This research did not receive any specific grant from any funding agency in the public, commercial or not-for-profit sector.

## References

[1] Selye H. The Stress of Life, 2nd ed. New York: McGraw-Hill, 1978.

[2] Lazarus RS Folkman S. Stress, Appriasal and Coping. New York: Springer Publishing, 1984.

[3] Ursin H Eriksen HR. The cognitive activation theory of stress. Psychoneuroendocrinology 2004 29 567–592. (10.1016/S0306-4530(03)00091-X) 15041082

[4] Dhabhar FS. The short-term stress response – mother nature’s mechanism for enhancing protection and performance under conditions of threat, challenge, and opportunity. Frontiers in Neuroendocrinology 2018 49 175–192. (10.1016/j.yfrne.2018.03.004) 29596867 PMC5964013

[5] Biondi M Picardi A. Psychological stress and neuroendocrine function in humans: the last two decades of research. Psychotherapy and Psychosomatics 1999 68 114–150. (10.1159/000012323) 10224513

[6] Campbell J Ehlert U. Acute psychosocial stress: does the emotional stress response correspond with physiological responses? Psychoneuroendocrinology 2012 37 1111–1134. (10.1016/j.psyneuen.2011.12.010) 22260938

[7] Bellingrath S Weigl T Kudielka BM. Chronic work stress and exhaustion is associated with higher allostastic load in female school teachers. Stress 2009 12 37–48. (10.1080/10253890802042041) 18951244

[8] Bonde JP. Psychosocial factors at work and risk of depression: a systematic review of the epidemiological evidence. Occupational and Environmental Medicine 2008 65 438–445. (10.1136/oem.2007.038430) 18417557

[9] Kivimaki M Virtanen M Elovainio M Kouvonen A Vaananen A Vahtera J. Work stress in the etiology of coronary heart disease – a meta-analysis. Scandinavian Journal of Work, Environment and Health 2006 32 431–442. (10.5271/sjweh.1049) 17173200

[10] Gerber M Jonsdottir IH Lindwall M Ahlborg GJ. Physical activity in employees with differing occupational stress and mental health profiles: a latent profile analysis. Psychology of Sport and Exercise 2014 15 649–658. (10.1016/j.psychsport.2014.07.012)

[11] Freudenberger HJ. Staff burn-out. Journal of Social Issues 1974 30 154–165.

[12] Maslach C Burned-out. Human Behaviour 1976 5 16–22.

[13] Maslach C Schaufeli WB Leiter MP. Job burnout. Annual Review of Psychology 2001 52 397–422. 10.1146/annurev.psych.52.1.397 11148311

[14] Shirom A. Burnout in work organization. In International Review of Industrial and Organizational Psychology. Eds CL Cooper & I Robertson New York: Wiley, 1989.

[15] Pines AM. Burnout: an existential perspective. In Professional Burnout: Recent Developments in Theory and Research, pp 1–16. Eds WB Schaufeli, C Maslach & T Marek Washington, DC: Taylor & Francis, 1993.

[16] Shirom A. Job-related burnout: a review. In Handbook of Occupational Health Psychology, pp 245–265. Eds C Quick & LE Tetrick Washington, DC: American Psychological Association, 2003.

[17] Schaufeli WB Leiter MP Maslach C. Burnout: 35 years of research and practice. Career Development International 2009 14 204–220. (10.1108/13620430910966406)

[18] Gustafsson H DeFreese JD Madigan DJ. Athlete burnout: review and recommendations. Current Opinion in Psychology 2017 16 109–113. (10.1016/j.copsyc.2017.05.002) 28813331

[19] Lindström C Åman J Norberg A. Increased prevalence of burnout symptoms in parents of chronically ill children. Acta Paediatrica, International Journal of Paediatrics 2010 99 427–432. (10.1111/j.1651-2227.2009.01586.x) 19912139

[20] Klarić M Frančišković T Pernar M Moro IN Milićević R Obrdalj EC Satriano AS. Caregiver burden and burnout in partners of war veterans with post-traumatic stress disorder. Collegium Antropologicum 2010 34 (Supplement 1) 15–21. 20402290

[21] Hasselberg K Jonsdottir IH Ellbin S Skagert K. Self-reported stressors among patients with exhaustion disorder: an exploratory study of patient records. BMC Psychiatry 2014 14 66 (10.1186/1471-244X-14-66) 24592907 PMC3975849

[22] Melamed S Kushnir T Shirom A. Burnout and risk factors for cardiovascular diseases. Behavioral Medicine 1992 18 53–60. (10.1080/08964289.1992.9935172) 1392214

[23] Melamed S Shirom A Toker S Berliner S Shapira I. Burnout and risk of cardiovascular disease: evidence, possible causal paths, and promising research directions. Psychological Bulletin 2006 132 327–353. (10.1037/0033-2909.132.3.327) 16719565

[24] Kivimaki M Kawachi I. Work stress as a risk factor for cardiovascular disease. Current Cardiology Reports 2015 17 630 (10.1007/s11886-015-0630-8) 26238744 PMC4523692

[25] Cohen R Bavishi C Haider S Thankachen J Rozanski A. Meta-analysis of relation of vital exhaustion to cardiovascular disease events. American Journal of Cardiology 2017 119 1211–1216. (10.1016/j.amjcard.2017.01.009) 28215416

[26] Toker S Melamed S Berliner S Zeltser D Shapira I. Burnout and risk of coronary heart disease: a prospective study of 8838 employees. Psychosomatic Medicine 2012 74 840–847. (10.1097/PSY.0b013e31826c3174) 23006431

[27] Appels A Schouten E. Burnout as a risk factor for coronary heart disease. Behavioral Medicine 1991 17 53–58. (10.1080/08964289.1991.9935158) 1878609

[28] Salvagioni DAJ Melanda FN Mesas AE González AD Gabani FL De Andrade SM. Physical, psychological and occupational consequences of job burnout: a systematic review of prospective studies. PLoS ONE 2017 12 e0185781 (10.1371/journal.pone.0185781) 28977041 PMC5627926

[29] Paine WS. Job Stress and Burnout: Research, Theory, and Intervention Perspectives. California, USA: Sage Publications, 1982.

[30] Schaufeli WB Bakker AB Hoogduin K Schaap C Kladler A. On the clinical validity of the Maslach burnout inventory and the burnout measure. Psychology and Health 2001 16 565–82. (10.1080/08870440108405527) 22804499

[31] Grossi G Perski A Osika W Savic I. Stress-related exhaustion disorder – clinical manifestation of burnout? A review of assessment methods, sleep impairments, cognitive disturbances, and neuro-biological and physiological changes in clinical burnout. Scandinavian Journal of Psychology 2015 56 626–636. (10.1111/sjop.12251) 26496458

[32] Kleijweg JHM Verbraak MJPM Van Dijk MK Comparing the predictive utility of two screening tools for mental disorder among probationers. Psychological Assessment 2013 25 435–441. (10.1037/a0031334) 23244643

[33] Glise K Ahlborg G Jonsdottir I. Course of mental symptoms in patients with stress-related exhaustion: does sex or age make a difference? BMC Psychiatry 2012 12 18 (10.1186/1471-244X-12-18) 22409935 PMC3338076

[34] Jonsdottir IH Hägg DA Glise K Ekman R. Monocyte chemotactic protein-1 (MCP-1) and growth factors called into question as markers of prolonged psychosocial stress. PLoS ONE 2009 4 e7659 (10.1371/journal.pone.0007659) 19888340 PMC2766003

[35] Danhof-Pont MB van Veen T Zitman FG. Biomarkers in burnout: a systematic review. Journal of Psychosomatic Research 2011 70 505–524. (10.1016/j.jpsychores.2010.10.012) 21624574

[36] Mommersteeg PMC Heijnen CJ Kavelaars A van Doornen LJP. Immune and endocrine function in burnout syndrome. Psychosomatic Medicine 2006 68 879–886. (10.1097/01.psy.0000239247.47581.0c) 17079708

[37] Mommersteeg PMC Heijnen CJ Verbraak MJPM van Doornen LJP. Clinical burnout is not reflected in the cortisol awakening response, the day-curve or the response to a low-dose dexamethasone suppression test. Psychoneuroendocrinology 2006 31 216–225. (10.1016/j.psyneuen.2005.07.003) 16150550

[38] Österberg K Karlson B Hansen ÅM. Cognitive performance in patients with burnout, in relation to diurnal salivary cortisol. Stress 2009 12 70–81. 18951245 10.1080/10253890802049699

[39] Sjörs A Ljung T Jonsdottir IH. Long-term follow-up of cortisol awakening response in patients treated for stress-related exhaustion. BMJ Open 2012 2 e001091. 10.1136/bmjopen-2012-001091 PMC3400075 22786949

[40] Grossi G Perski A Ekstedt M Johansson T Lindström M Holm K. The morning salivary cortisol response in burnout. Journal of Psychosomatic Research 2005 59 103–111. (10.1016/j.jpsychores.2005.02.009) 16186006

[41] Sjörs A Jonsdottir IH. No alterations in diurnal cortisol profiles before and during the treatment in patients with stress-related exhaustion. International Journal of Occupational Medicine and Environmental Health 2015 28 120–129. (10.13075/ijomeh.1896.00208) 26159953

[42] De Vente W Olff M Van Amsterdam JGC Kamphuis JH Emmelkamp PMG. Physiological differences between burnout patients and healthy controls: blood pressure, heart rate, and cortisol responses. Occupational and Environmental Medicine 2003 60 (Supplement 1) i54–i61. (10.1136/oem.60.suppl_1.i54) 12782748 PMC1765727

[43] Mommersteeg PMC Heijnen CJ Keijsers GPJ Verbraak MJPM Van Doornen LJP. Cortisol deviations in people with burnout before and after psychotherapy: a pilot study. Health Psychology 2006 25 243–248. (10.1037/0278-6133.25.2.243) 16569117

[44] Stalder T Kirschbaum C Kudielka BM Adam EK Pruessner JC Wust S Dockray S Smyth N Evans P Hellhammer DH ***et al*** Assessment of the cortisol awakening response: expert consensus guidelines. Psychoneuroendocrinology 2016 63 414–432. (10.1016/j.psyneuen.2015.10.010) 26563991

[45] Elder GJ Wetherell MA Barclay NL Ellis JG. The cortisol awakening response – applications and implications for sleep medicine. Sleep Medicine Reviews 2014 18 215–224. (10.1016/j.smrv.2013.05.001) 23835138

[46] Bellingrath S Weigl T Kudielka BM. Cortisol dysregulation in school teachers in relation to burnout, vital exhaustion, and effort-reward-imbalance. Biological Psychology 2008 78 104–113. (10.1016/j.biopsycho.2008.01.006) 18325655

[47] Söderström M Ekstedt M Åkerstedt T. Weekday and weekend patterns of diurnal cortisol, activation and fatigue among people scoring high for burnout. Scandinavian Journal of Work, Environment and Health 2006 2 (Supplement) 35–40.

[48] Marchand A Durand P Juster R-P Lupien SJ. Workers’ psychological distress, depression, and burnout symptoms: associations with diurnal cortisol profiles. Scandinavian Journal of Work, Environment and Health 2014 40 305–314. (10.5271/sjweh.3417) 24469265

[49] Pilger A Haslacher H Meyer BM Lackner A Nassan-Agha S Nistler S Stangelmaier C Endler G Mikulits A Priemer I ***et al*** Midday and nadir salivary cortisol appear superior to cortisol awakening response in burnout assessment and monitoring. Scientific Reports 2018 8 9151 (10.1038/s41598-018-27386-1) 29904183 PMC6002544

[50] Wingenfeld K Schulz M Damkroeger A Rose M Driessen M. Elevated diurnal salivary cortisol in nurses is associated with burnout but not with vital exhaustion. Psychoneuroendocrinology 2009 34 1144–1151. (10.1016/j.psyneuen.2009.02.015) 19321266

[51] Melamed S Ugarten U Shirom A Kahana L Lerman Y Froom P. Chronic burnout, somatic arousal and elevated salivary cortisol levels. Journal of Psychosomatic Research 1999 46 591–598. (10.1016/S0022-3999(99)00007-0) 10454175

[52] Oosterholt BG Maes JHR Van der Linden D Verbraak MJPM Kompier MAJ. Burnout and cortisol: evidence for a lower cortisol awakening response in both clinical and non-clinical burnout. Journal of Psychosomatic Research 2015 78 445–451. (10.1016/j.jpsychores.2014.11.003) 25433974

[53] Ekstedt M Akerstedt T Soderstrom M. Microarousals during sleep are associated with increased levels of lipids, cortisol, and blood pressure. Psychosomatic Medicine 2004 66 925–931. (10.1097/01.psy.0000145821.25453.f7) 15564359

[54] Onen Sertoz O Tolga Binbay I Koylu E Noyan A YildIrIm E Elbi Mete H. The role of BDNF and HPA axis in the neurobiology of burnout syndrome. Progress in Neuro-Psychopharmacology and Biological Psychiatry 2008 32 1459–1465. (10.1016/j.pnpbp.2008.05.001) 18541357

[55] Rydmark I Wahlberg K Ghatan PH Modell S Nygren Å Ingvar M Åsberg M Heilig M. Neuroendocrine, cognitive and structural imaging characteristics of women on longterm sickleave with job stress-induced depression. Biological Psychiatry 2006 60 867–873. (10.1016/j.biopsych.2006.04.029) 16934773

[56] Wahlberg K Ghatan PH Modell S Nygren Å Ingvar M Åsberg M Heilig M. Suppressed neuroendocrine stress response in depressed women on job-stress-related long-term sick leave: a stable marker potentially suggestive of preexisting vulnerability. Biological Psychiatry 2009 65 742–747. (10.1016/j.biopsych.2008.10.035) 19058782 PMC2745651

[57] Pruessner JC Hellhammer DH Kirschbaum C. Burnout, perceived stress, and cortisol responses to awakening. Psychosomatic Medicine 1999 61 197–204. (10.1097/00006842-199903000-00012) 10204973

[58] Wolfram M Bellingrath S Feuerhahn N Kudielka BM. Emotional exhaustion and overcommitment to work are differentially associated with hypothalamus-pituitary-adrenal (HPA) axis responses to a low-dose ACTH1–24 (Synacthen) and dexamethasone-CRH test in healthy school teachers. Stress 2013 16 54–64. (10.3109/10253890.2012.683465) 22564145

[59] de Vente W van Amsterdam JGC Olff M Kamphuis JH Emmelkamp PMG. Burnout is associated with reduced parasympathetic activity and reduced HPA axis responsiveness, predominantly in males. BioMed Research International 2015 2015 13. 10.1155/2015/431725 PMC4628754 26557670

[60] Lennartsson AK Sjors A Wahrborg P Ljung T Jonsdottir IH. Burnout and hypocortisolism – a matter of severity? A study on ACTH and cortisol responses to acute psychosocial stress. Frontiers in Psychiatry 2015 6 8 (10.3389/fpsyt.2015.00008) 25698980 PMC4313581

[61] Jönsson P Österberg K Wallergård M Hansen ÅM Garde AH Johansson G Karlson B. Exhaustion-related changes in cardiovascular and cortisol reactivity to acute psychosocial stress. Physiology and Behavior 2015 151 327–337. (10.1016/j.physbeh.2015.07.020) 26210042

[62] Cadegiani FA Kater CE. Adrenal fatigue does not exist: a systematic review. BMC Endocrine Disorders 2016 16 48 (10.1186/s12902-016-0128-4) 27557747 PMC4997656

[63] Langelaan S Bakker AB Schaufeli WB van Rhenen W van Doornen LJP. Do burned-out and work-engaged employees differ in the functioning of the hypothalamic-pituitary-adrenal axis? Scandinavian Journal of Work, Environment and Health 2006 32 339–348. (10.5271/sjweh.1029) 17091201

[64] Moch S Panz V Joffe B Havlik I Moch J. Longitudinal changes in pituitary-adrenal hormones in south african women with burnout. Endocrine 2003 21 267–272. (10.1385/ENDO:21:3:267) 14515012

[65] Morgan CA III Cho T Hazlett G Coric V Morgan J. The impact of burnout on human physiology and on operational performance: a prospective study of soldiers enrolled in the combat diver qualification course. Yale Journal of Biology and Medicine 2002 75 199–205. 12784969 PMC2588792

[66] Grossi G Perski A Evengård B Blomkvist V Orth-Gomér K. Physiological correlates of burnout among women. Journal of Psychosomatic Research 2003 55 309–316. (10.1016/S0022-3999(02)00633-5) 14507541

[67] Tops M Boksem MAS Wijers AA van Duinen H Den Boer JA Meijman TF Korf J. The psychobiology of burnout: are there two different syndromes? Neuropsychobiology 2007 55 143–150. (10.1159/000106056) 17641533

[68] Dahlgren A Kecklund G Theorell T Åkerstedt T. Day-to-day variation in saliva cortisol – relation with sleep, stress and self-rated health. Biological Psychology 2009 82 149–155. (10.1016/j.biopsycho.2009.07.001) 19596045

[69] McEwen BS. Protective and damaging effects of stress mediators: central role of the brain. Dialogues in Clinical Neuroscience 2006 8 367–381. 17290796 10.31887/DCNS.2006.8.4/bmcewen PMC3181832

[70] Maninger N Wolkowitz OM Reus VI Epel ES Mellon SH. Neurobiological and neuropsychiatric effects of dehydroepiandrosterone (DHEA) and DHEA sulfate (DHEAS). Frontiers in Neuroendocrinology 2009 30 65–91. (10.1016/j.yfrne.2008.11.002) 19063914 PMC2725024

[71] Kalimi M Shafagoj Y Loria R Padgett D Regelson W. Anti-glucocorticoid effects of dehydroepiandrosterone (DHEA). Molecular and Cellular Biochemistry 1994 131 99–104. (10.1007/BF00925945) 8035785

[72] Morgan Iii CA Southwick S Hazlett G Rasmusson A Hoyt G Zimolo Z Charney D. Relationships among plasma dehydroepiandrosterone sulfate and cortisol levels, symptoms of dissociation, and objective performance in humans exposed to acute stress. Archives of General Psychiatry 2004 61 819–825. (10.1001/archpsyc.61.8.819) 15289280

[73] Lennartsson AK Kushnir MM Bergquist J Jonsdottir IH. DHEA and DHEA-S response to acute psychosocial stress in healthy men and women. Biological Psychology 2012 90 143–149. (10.1016/j.biopsycho.2012.03.003) 22445967

[74] Lennartsson AK Theorell T Kushnir MM Jonsdottir IH Meijer OC. Low levels of dehydroepiandrosterone sulfate in younger burnout patients. PLoS ONE 2015 10 e0140054 (10.1371/journal.pone.0140054) 26441131 PMC4595129

[75] Lennartsson A-K Sjörs A Jonsdottir IH. Indication of attenuated DHEA-s response during acute psychosocial stress in patients with clinical burnout. Journal of Psychosomatic Research 2015 79 107–111. (10.1016/j.jpsychores.2015.05.011) 26071787

[76] Lennartsson AK Theorell T Kushnir MM Jonsdottir IH. Changes in DHEA-s levels during the first year of treatment in patients with clinical burnout are related to health development. Biological Psychology 2016 120 28–34. (10.1016/j.biopsycho.2016.08.003) 27531310

[77] Åsberg M Nygren Å Leopardi R Rylander G Peterson U Wilczek L Källmén H Ekstedt M Åkerstedt T Lekander M ***et al*** Novel biochemical markers of psychosocial stress in women. PLoS ONE 2009 4 1–5. 10.1371/journal.pone.0003590 PMC2627901 19177163

[78] Lennartsson A-K Billig H Jonsdottir IH. Burnout is associated with elevated prolactin levels in men but not in women. Journal of Psychosomatic Research 2014 76 380–383. (10.1016/j.jpsychores.2014.03.007) 24745779

[79] Aguilera G. Chapter 8 – The hypothalamic–pituitary–adrenal axis and neuroendocrine responses to stress. In Handbook of Neuroendocrinology, pp 175–196. Eds G Fink, DW Pfaff & JE Levine San Diego: Academic Press, 2012.

[80] Nadolnik L. Stress and the thyroid gland. Biochemistry Supplemental Series B: Biomedical Chemistry 2011 5 103–112. (10.1134/S1990750811020119)

[81] Guo Y Lam L Luo Y Plummer V Cross W Li H Yin Y Zhang J. Female nurses’ burnout symptoms: No association with the hypothalamic-pituitary-thyroid (HPT) axis. Psychoneuroendocrinology 2017 77 47–50. (10.1016/j.psyneuen.2016.11.020) 28012293

[82] Metlaine A Sauvet F Gomez-Merino D Boucher T Elbaz M Delafosse JY Leger D Chennaoui M. Sleep and biological parameters in professional burnout: a psychophysiological characterization. PLoS ONE 2018 13 e0190607 (10.1371/journal.pone.0190607) 29385150 PMC5791983

[83] Drago F D'Agata V Iacona T Spadaro F Grassi M Valerio C Astuto C Lauria N Raffaele R Vitetta M. Prolactin as a protective factor in stress‐induced biological changes. Journal of Clinical Laboratory Analysis 1989 3 340–344. (10.1002/jcla.1860030605) 2693666

[84] Dorshkind K Horseman ND Anterior pituitary hormones, stress, and immune system homeostasis. BioEssays 2001 23 288–294. (10.1002/1521-1878(200103)23:3<288::AID-BIES1039>3.0.CO;2-P) 11223886

[85] Kiecolt-Glaser JK McGuire L Robles TF Glaser R. Psychoneuroimmunology and psychosomatic medicine: back to the future. Psychosomatic Medicine 2002 64 15–28. (10.1097/00006842-200201000-00004) 11818582

[86] Johnson TV Abbasi A Master VA. Systematic review of the evidence of a relationship between chronic psychosocial stress and C-reactive protein. Molecular Diagnosis and Therapy 2013 17 147–164. (10.1007/s40291-013-0026-7) 23615944

[87] Langelaan S Schaufeli W van Doornen L Bakker A van Rhenen W. Is burnout related to allostatic load? International Journal of Behavioral Medicine 2007 14 213–221. (10.1007/BF03002995) 18001236

[88] Toker S Shirom A Shapira I Berliner S Melamed S. The association between burnout, depression, anxiety, and inflammation biomarkers: C-reactive protein and fibrinogen in men and women. Journal of Occupational Health Psychology 2005 10 344–362. (10.1037/1076-8998.10.4.344) 16248685

[89] Bargellini A Barbieri A Rovesti S Vivoli R Roncaglia R Borella P. Relation between immune variables and burnout in a sample of physicians. Occupational and Environmental Medicine 2000 57 453–457. (10.1136/oem.57.7.453) 10854497 PMC1739992

[90] Gajewski PD Boden S Freude G Potter GG Claus M Bröde P Watzl C Getzmann S Falkenstein M. Executive control, ERP and pro-inflammatory activity in emotionally exhausted middle-aged employees. Comparison between subclinical burnout and mild to moderate depression. Psychoneuroendocrinology 2017 86 176–186. (10.1016/j.psyneuen.2017.09.017) 28972891

[91] Dragos D Tanasescu MD. The effect of stress on the defense systems. Journal of Medicine and Life 2010 3 10–18. 20302192 PMC3019042

[92] Nakamura H Nagase H Yoshida M Ogino K. Natural killer (Nk) Cell activity and nk cell subsets in workers with a tendency of burnout. Journal of Psychosomatic Research 1999 46 569–578. (10.1016/S0022-3999(99)00009-4) 10454173

[93] von Känel R Bellingrath S Kudielka BM. Association between burnout and circulating levels of pro- and anti-inflammatory cytokines in schoolteachers. Journal of Psychosomatic Research 2008 65 51–59. (10.1016/j.jpsychores.2008.02.007) 18582612

[94] Shirom A Ezrachi Y. On the discriminant validity of burnout, depression and anxiety: a re-examination of the burnout measure. Anxiety, Stress, and Coping 2003 16 83–97. (10.1080/1061580021000057059)

[95] Gelman A Carlin J. Beyond power calculations:assessing type S (sign) and type M (magnitude) errors. Perspectives on Psychological Science 2014 9 641–651. (10.1177/1745691614551642) 26186114

